# The Registration of Midwives and Nurses

**Published:** 1889-12-14

**Authors:** 


### The Registration of Midwives and Nurses.

### IMPORTANT DECISION OF THE GENERAL COUNCIL OF MEDICAL EDUCATION AND REGISTRATION.

The recent session of the General Council of Medical Education must have a very important bearing on the proposed registration of midwives and nurses. At the meetings of the council on 26th and 29th ult., Prof. Marshall, F.R.S. (president), in the chair, communications in reference to the registration of midwives—comprising a correspondence between the president and Mr. Fell Pease, M.P., representing the Midwive's Institute, duly incorporated, on behalf of which body he will introduce a Bill into Parliament in February next, and a memorandum addressed to the council by the British Nurses' Association—were received, entered on the minutes, and debated.

In order to understand these proceedings, it must be remembered that the Midwive's Institute has for many years consistently laboured to secure adequate protection to the poor by passing an Act to provide for the registration and improved education of midwives. It is now an incorporated Institute and has a reserve fund of £1,000. The Midwive's Institute has always maintained that midwives must be held to be on an altogether different footing to nurses, and that no experienced persons possessed of adequate technical knowledge could consistently advocate a common system of registration for two groups of women so entirely separated by the circumstances of their training, education, and surroundings as midwives and nurses. The British Nurses' Association, formed at the end of 1887, advocated the granting of a charter under which powers were to be conferred upon its council "to appoint a registration board for nurses and midwives." This charter has not been proceeded with, and is, indeed, reported to have been abandoned. Although the British Nurses' Association thus gave up the charter, its council determined to commence a voluntary system of registration for nurses and midwives, and the memorandum referred to above, and the speeches of the supporters of the British' Nurses Association in the Medical Council, were in effect in support of the recognition of this system as opposed to that of the Midwive's Institute.

It will be seen that the General Medical Council rejected the amendment of Mr. Brudenell Carter, a member of the council of the British Nurses' Association, who moved that the word "local" should be omitted from Sir John Simon's resolution, and that it should include "charter" and "the registration of nurses" as well as midwives. The scheme of the British Nurses' Association was thus practically submitted to the judgment of the General Medical Council, and, on a division, was rejected. Indeed, Dr. McAlister, in seconding Mr. Carter's amendment, distinctly asked the council not to reject the amendment, as by so doing they would "cast off the other," that is, the British Nurses' Association.

We have decided to reproduce the following report of the discussion at the General Medical Council on this subject from the *Lancet*, in preference to giving our own independent report, because that paper has always favoured the British Nurses' Association, and the report may therefore be taken not to unduly favour the case of its opponents:

### Licensing and Registration of Midwives.

Sir John Simon moved: "If the Local Government Board or any other department of Her Majesty's Government were constituted controlling authority in relation to the local arrangements which might be made under statute for the licensing and registration of midwives, the Medical Council would, if Government so wished, be willing to advise as to the general rules of education, examination, and discipline which ought to be established in the matter; but the council would not be able to discharge, and would, therefore, not be prepared to undertake any duties of detail as to the registration of midwives, or as to the local arrangements for licensing and controlling them." He said the great object in dealing with the matter was to maintain continuity with former proceedings of the council. This subject was not a new one. As far back as 1877 certain regulations were passed by the council in reference to the midwifery question, and those regulations were in the following year confirmed. In 1882 a committee, of which the President was chairman, further dealt with the matter, and in the main the recommendations of that committee were approved by the council—viz., that there was great need of a good national system of dealing with midwives, and that the council was ready to give its assistance in a particular way if required. The resolution of 1877 was: "The council is of opinion that it would be desirable to provide by legislation concerning two objects—first, the means under legal sanction which would be provided for giving credentials to competent midwives; and, secondly, that the lives of women in labour should, so far as practicable, be protected from the incompetent." That resolution was communicated to the Lord President of the Council. In 1878 the opinion was expressed by the council that it was not necessary that a central registry of midwives should be kept, but that there should be local registers, and the duty of keeping them should devolve upon the local authorities in the centres where the examinations were conducted. In 1882 both those opinions were confirmed by the council. That was their present position, and the resolution he brought forward was intended to define how far they were prepaied to go. He was sure every member would agree that the additional work thrown upon them for the last few years made it even more open to question than it was in 1877-78 whether the council should undertake local obligations in this matter.

Sir Walter Foster seconded the motion. He said he had been induced to give notice of a resolution on this question by what he felt was the growing scandal to civilisation, that they should have incompetent persons taking charge of women under perilous conditions, and should have a considerable amount of disease brought about by their ignorance. The very last case of puerperal fever which he had seen was found to have arisen from the nurse having occasionally left her patient to go into another house to attend a child suffering from a malignant form of sore throat. This showed how the ignorance of midwives was really a peril to the community, and that peril falling upon the poorest classes, it was a matter of public interest that some steps should be taken to prevent it. To that end it was essential that these women should be educated in some form, and that when educated they should be under some supervision and restraint. Some objection might be raised with reference to the authority that it was proposed to invoke. They did not propose any local authority, but would rather that it should be under the control of a central authority such as the Local Government Board. It would, however, be necessary that the Local Government Board should constitute some local authority to carry out this registration. Such a proceeding would have this advantage, that the public would then be able to find out at once whether a person acting as midwife had been properly registered and possessed the necessary qualifications to protect them from incompetence.

Dr. Atthill said that, owing to his position as master of the Rotunda Hospital, his experience in relation to midwives was necessarily very great. The Rotunda Hospital licensed annually a large number of women, and the greatest possible care was taken that those women were qualified. They had to remain at the hospital for six months, during which time they had an opportunity of seeing certainly not less than a thousand deliveries. They were lectured regularly by the assistants and sometimes by the master, and finally they passed an examination of very fair stringency. Notwithstanding all this, it was very hard to get a good midwife. The great majority of the applicants were servants, who, having been for perhaps twenty years in service, got tired of that occupation, and came up for training, the fees in some cases being paid by their former mistresses. Some turned out fairly well, but a great many were merely second-rate. In addition to that, there were women sent up from all parts of the country, some by the Poor Law guardians, to be educated for their particular districts. This was the worst class of all, many of them not being able either to read or write. If this was the experience in Ireland, what would it be in the country at large where there was no such system as had for years been in existence in Ireland? Some of those who came to the Rotunda were well-educated women who wished to make it a profession, and they turned out exceedingly competent. The great difficulty had always been to get competent midwives for the country districts, although the pay they received in Ireland was greater than it was in England. He was afraid that any attempt to carry out such a scheme as was proposed would be attended with great difficulty.

Mr. Brudenell Carter said it was tolerably notorious that there were two agencies at work covering pretty much the same ground, with this marked distinction, that one was entirely under the control of medical practitioners and trained nurses, whereas the other was partially under the control of non-medical persons. Under these circumstances it was desirable that any resolution of the council should be so framed as to leave an open door for some authoritative supervision of the education and registration of trained nurses, as well as of midwives, and not to tie itself down to some local arrangement which might or might not be desirable. No doubt if the whole matter were remitted to the council by the authorities, its inclination would be to support a purely medical association in connection with these matters. He proposed as an amendment that the word "local" should be omitted from the resolution, and that it should include the registration of nurses as well as midwives. It would then afford somewhat greater scope for useful action in the future.

Sir John Simon thought the two subjects had better not be mixed up together. The subject of midwives was forced upon them by Parliament, and was ripe for legislation, but the other was not. He could not accept the amendment.

Dr. Atthill was in favour of the two words being used, so that everyone should be licensed as midwife and nurse. His reason was that they found in Ireland that if women were licensed as midwives only they immediately assumed the position of practitioners. The license was now given as "midwife and nurse."

Dr. Glover expressed his extreme gratification that this subject had been raised by so eminent a medical statesman as Sir John Simon. He objected, however, to the authority being handed over to the Local Government Board, which was associated with all sorts of political considerations, so that there was no saying what elements might be introduced into the question if that particular department was approached. Another objection was that the County Council would be entirely guided in the first instance by the medical officer of health. He was a most important person, and destined to be more so in future, but he should not have so predominating an authority as Sir John Simon's resolution would give him. He hoped that the very prominent suggestion of the Local Government Board would not be pressed, as he was sure it would not be acceptable.

Sir John Simon said they must recognise the constitution of the country, and if the Local Government Board was the department which looked after matters of local administration, that fact must be recognised. He perhaps had as little reason as most people to have sympathy with the Local Government Board, but the state of the law was that that was the body superior to county authorities. He did not name the county authorities in the resolution, and in mentioning the Local Government Board he added "or any other department of Her Majesty's Government." With reference to the very valuable remarks made by Dr. Atthill, there could be no doubt what they meant by midwives, and it would be very easy in laying down rules for their examination to make such provision as was necessary for their relation to nursing. The introduction of the word "nursing" into this very general resolution would only embarrass it.

Mr. Wheelhouse seconded Dr. Glover's suggestion for the omission of the words "Local Government Board."

Dr. Leishman said, though he could not claim the extensive experience of Dr. Atthill with regard to the training of midwives, he had some experience in that direction, and was glad to state that matters were very much better all through the country than they were not many years ago. A good deal was being done by the agencies at work for which their gratitude was due; but there was a paramount necessity for something more. He gave his entire support to the resolution, and at once recognised Sir John Simon's plea that if they bracketed the question with that of nurses an element of difficulty would be introduced. One great difficulty was as to the sources from which midwives and nurses were obtained. No one would for a moment doubt that a nurse educated at the Rotunda Hospital would be a person deserving of confidence; but many women called themselves "certified nurses" who had received certificates from persons who had not the slightest warrant to grant them. The term must be made to mean something definite; that a woman had had a sufficient amount of training, and also elementary education, which would enable her to approach the subject with ordinary intelligence and a certain amount of practical experience. Any legislation recognising this fact could not fail to be beneficial.

Mr. Brudenell Carter submitted his amendment in the following form: "That if the Local Government Board, or any other department of Her Majesty's Government, were constituted controlling authority in relation to any arrangements made under statute or charter for the licensing and registration of midwives, or of midwives and nurses, the Medical Council would, if the Government department so wished, be willing to advise as to the general rules of education, examination, and discipline which ought to be established in the matter; but the council would not be able to discharge, and would, therefore, not be prepared to undertake, any duties of detail as to the registration of midwives and nurses, or as to the arrangements necessary for licensing and controlling them."

Dr. MacAlister seconded the amendment. He said there were two associations which he might call rivals, each desiring to be placed in control of nursing education. The British Nurses' Association was controlled by medical men, but the other was wholly without control. He thought the council should be extremely wary how it took up one of these and cast off the other; but that was what Sir John Simon's motion would do. Such a proceeding would be at once taken up for advertising purposes, and therefore he urged that any answer given by the council should be in words which would be applicable to both.

Sir John Simon said he never had the slightest notion of any rivalry between the societies. He wished to give every possible assistance to the nursing movement; but it was entirely separate from the question of the education and registration of midwives.

Sir Wm. Turner thought that in going into the question of nurses the council was exceeding its powers. It was a Council of Medical Education and Registration, not a nursing institution. He did not see why the council should mix up in a squabble between these two bodies.

The amendment was then put to the council and negatived.

Sir John Simon said, in reply to Dr. Glover, he had no objection to leave out the words "Local Government Board," and with the omission of these words, the resolution was put, and agreed to.

It was moved by Sir John Simon, seconded by Sir Walter Foster, and agreed to: "That, with reference to the communications which have passed between Mr. Pease and the president (pp. 144-147 of minutes for Nov. 26th), the president be authorised to inform Mr. Pease that the council regards with much regret the absence of proper public provision for the certification of competent midwives, and believes that the want of such provision is conducive to a large amount of suffering and danger to life among lying-in women of the poorer classes. That the president be requested to bring to Mr. Pease's knowledge the proceedings of the council in the years 1877, 1878, and 1882 in relation to midwives, especially the following resolutions: Vol. xiv., p. 198, secs. 14 and 15; vol. xv., p. 65, sec. 13; vol. xix., p. 106, sec. 13, and p. 108, *Ha*; and, further, to let Mr. Pease have a copy of the resolution this day passed by the council on the part which the council, if so desired by her Majesty's Government, would be ready to take in a national system for the regulation of midwives."

It was also proposed by Sir Walter Foster, seconded by Dr. Glover, and agreed to: "That this council regards the absence of public provision for the education and supervision of midwives as productive of a large amount of grave suffering and fatal disease among the poorer classes, and urges upon the Government the importance of passing into law some measure for the education and registration of midwives."

